# Responsiveness of Outcome Measures in Chronic Non-Specific Low Back Pain: A Secondary Analysis of a Randomized Controlled Trial

**DOI:** 10.3390/jpm16070338

**Published:** 2026-06-23

**Authors:** Carlos Luques Fonseca, Pedro Augusto Silva Ribeiro, Karla Cristina Naves de Carvalho, Rodrigo Antonio Carvalho Andraus, Renata Calhes Franco de Moura, Andrei Machado Viegas da Trindade, Arislander Jonathan Lopes Dumont, Tiago Vieira Fernandes, Daniel Grossi Marconi, Hugo Pasin Neto, Danilo Armbrust, Claudia Santos Oliveira

**Affiliations:** 1 Department of Physiotherapy, Faculty of Medical Sciences, Santa Casa de São Paulo, São Paulo 01221-020, SP, Brazilcsantos.neuro@gmail.com (C.S.O.); 2 School of Health Sciences, Evangelical University of Goiás, Anápolis 75083-515, GO, Brazilrodrigoandraus@gmail.com (R.A.C.A.); 3 School of Physiotherapy, University of Sorocaba, Sorocaba 18023-000, SP, Brazil; arislanderlg@gmail.com (A.J.L.D.);; 4 Brazilian College of Osteopathy, São Paulo 18040-280, SP, Brazil; 5 Department Rehabilitation, Barretos Cancer Hospital, Barretos 14784-400, SP, Brazil; 6 Department of Physiotherapy, Anhanguera College of Sorocaba, Sorocaba 18016-110, SP, Brazil; danilo.armbrust@anhanguera.com

**Keywords:** chronic non-specific low back pain, osteopathic manipulative treatment, transcranial direct current stimulation

## Abstract

**Background/Objectives:** Chronic non-specific low back pain (CNLBP) is a leading cause of disability worldwide. Although several randomized trials have evaluated treatment effectiveness, less attention has been given to the responsiveness of outcome measures used to assess clinical change. This study aimed to evaluate the internal and external responsiveness of commonly used outcome measures in individuals with CNLBP. **Methods:** This study is a secondary analysis of a randomized controlled trial. Participants were analyzed as active and placebo groups and assessed at baseline, post-intervention, and follow-up. Internal responsiveness was evaluated using standardized mean differences (SMD) and standardized response means (SRM). External responsiveness was assessed using anchor-based approaches, including correlations with the Global Rating of Change Scale (GRCS) and receiver operating characteristic (ROC) curve analysis. **Results:** Outcome measures demonstrated moderate to high internal responsiveness, with large effect sizes observed for pain intensity (NRS) and quality of life (EQ-5D-3L). However, external responsiveness was limited, with all instruments presenting area under the curve (AUC) values below 0.70. The Bournemouth Questionnaire showed the highest discriminative performance among the instruments. **Conclusions:** The evaluated instruments were sensitive to detecting change at the group level but showed limited ability to discriminate clinically meaningful improvement at the individual level. These findings support the use of combined outcome measures to improve clinical interpretation and decision-making in CNLBP.

## 1. Introduction

Low back pain (LBP) is defined as discomfort or musculoskeletal tension located between the lower margin of the ribs and the gluteal folds, with or without radiation to the lower limbs [1,2,3]. It is one of the most prevalent musculoskeletal conditions worldwide and remains the leading cause of years lived with disability over the past decades [1,4].

Approximately 90–95% of cases are classified as non-specific low back pain, in which no identifiable pathological cause can be established [2,5]. Chronic non-specific low back pain (CNLBP), defined as symptoms lasting longer than 12 weeks, is currently recognized as a primary chronic pain condition and is frequently associated with nociplastic mechanisms and alterations in central nervous system processing [6,7].

Evidence indicates that CNLBP is associated with structural, functional, and neurochemical changes in brain regions involved in pain processing, including the prefrontal cortex, insula, cingulate cortex, and thalamus [7,8,9]. These findings reinforce the multifactorial nature of the condition, which involves complex biological, psychological, and social factors [10].

Osteopathic manipulative treatment (OMT) has demonstrated clinically relevant effects in reducing pain intensity and improving functional outcomes in individuals with CNLBP [11,12]. Transcranial direct current stimulation (tDCS), a non-invasive neuromodulation technique, has been investigated for its ability to modulate cortical excitability and pain-related neural networks; however, its clinical effectiveness remains inconsistent [13,14].

Despite the growing number of randomized controlled trials evaluating therapeutic interventions for CNLBP, less attention has been given to the measurement properties of the instruments used to assess clinical outcomes. Responsiveness, defined as the ability of an instrument to detect change over time, is a key component of longitudinal validity and is essential for interpreting treatment effectiveness [15].

Responsiveness can be examined through two complementary approaches: internal responsiveness, which reflects the magnitude of change detected by an instrument over time, and external responsiveness, which represents the relationship between score changes and an external criterion of clinical importance, such as patient-perceived improvement [5,15].

Commonly used outcome measures in CNLBP, including the Numeric Rating Scale (NRS), Roland–Morris Disability Questionnaire (RMDQ), and EQ-5D-3L, have demonstrated adequate measurement properties; however, their ability to discriminate clinically meaningful change at the individual level remains uncertain [7,8].

Previous studies evaluating responsiveness in low back pain populations have reported heterogeneous findings, with variability depending on the selected instruments and methodological approaches. However, few studies have simultaneously compared multiple commonly used outcome measures using both internal and external responsiveness within the same clinical sample derived from a randomized controlled trial.

Therefore, the evaluation of responsiveness using data derived from randomized controlled trials may provide a more robust understanding of the performance of clinical instruments in real-world conditions.

The aim of this study was to evaluate the internal and external responsiveness of commonly used outcome measures in individuals with chronic non-specific low back pain using data from a randomized controlled trial.

## 2. Materials and Methods

### 2.1. Study Design

This study is a secondary analysis of data derived from a previously published randomized controlled trial investigating the effects of osteopathic manipulative treatment (OMT) combined with transcranial direct current stimulation (tDCS) in individuals with chronic non-specific low back pain.

The original trial was a prospective, randomized, placebo-controlled, double-blind study with three parallel groups. In contrast, the present study aimed to evaluate the internal and external responsiveness of outcome measures. For this purpose, the original three-arm randomized controlled trial was collapsed into two conditions (active and placebo).

This analytical approach was adopted to increase statistical power for responsiveness analyses and to prioritize the evaluation of measurement properties rather than between-group treatment effects. However, this strategy may limit the interpretation of treatment-specific effects and should be considered when interpreting the findings. This approach is commonly used in studies investigating measurement properties.

### 2.2. Ethical Considerations

This study is a secondary analysis of a randomized controlled trial that was prospectively registered in the Brazilian Clinical Trials Registry (ReBEC) (ID: RBR-3X5PGN7) on 16 December 2020. The research protocol was initially submitted for ethical approval in 2020 and approved by the Research Ethics Committee of Irmandade da Santa Casa de Misericórdia de São Paulo (CAAE: 38113920.2.0000.5479). A protocol amendment (Version 3) was approved on 15 December 2022. Participants were recruited between May 2022 and July 2023.

### 2.3. Participants

Participants were recruited from the original randomized controlled trial and included adults aged 18–55 years diagnosed with chronic non-specific low back pain (pain duration ≥ 12 weeks). Only participants with complete data across all assessment time points (T0, T1, and T2) were included in this analysis. A priori sample size calculation was not performed for this secondary analysis, as all available data from the original trial were included.

### 2.4. Study Timeline and Assessments

Assessments were conducted at baseline (T0), post-intervention after two weeks (T1), and follow-up one month after treatment completion (T2).

### 2.5. Outcome Measures

Outcome measures were selected to evaluate the responsiveness of instruments assessing pain, functional disability, biopsychosocial impact, and quality of life. Pain intensity was assessed using the Numeric Rating Scale (NRS; 0–10). Functional disability was evaluated using the Roland–Morris Disability Questionnaire (RMDQ; 0–24) and the Oswestry Disability Index (ODI; 0–100%). Biopsychosocial factors were assessed using the Bournemouth Questionnaire. Quality of life was assessed using the EQ-5D-3L.

External responsiveness was evaluated using the Global Rating of Change Scale (GRCS), ranging from −7 to +7. Participants were classified as improved (≥+2) or not improved (<+2), and this classification was used as an anchor.

### 2.6. Responsiveness Analysis

Internal responsiveness was evaluated using distribution-based methods, including standardized mean difference (SMD) and standardized response mean (SRM). External responsiveness was assessed using anchor-based methods, including correlations with the Global Rating of Change Scale (GRCS) and ROC curve analysis. A cut-off point of ≥+2 on the GRCS was used to define clinically meaningful improvement. This threshold has been commonly adopted in previous studies and reflects a perceptible and relevant change from the patient’s perspective. However, it is acknowledged that different cut-off values may influence responsiveness estimates and classification accuracy.

### 2.7. Intervention Description

Intervention procedures were conducted as described in the original trial. Briefly, tDCS was delivered using a direct current stimulator (NeuroConn DC-Stimulator Plus, neuroCare Group, Ilmenau, Germany) with a current intensity of 2 mA for 20 minutes over two weeks, targeting the primary motor cortex. OMT was delivered in two sessions using individualized techniques. Sham procedures were applied to maintain participant blinding.

### 2.8. Statistical Analysis

Statistical analyses were performed using SPSS software (version 19.0, IBM Corp., Armonk, NY, USA). Descriptive statistics were calculated for all variables. Internal responsiveness indices (SMD and SRM) were computed for each outcome. External responsiveness was evaluated using ROC curves and correlation analyses. AUC values were interpreted as follows: <0.70, low accuracy; 0.70–0.80, acceptable; 0.80–0.90, excellent; >0.90, outstanding. Statistical significance was set at *p* < 0.05.

### 2.9. Relationship with Previous Publication

This study represents a secondary analysis of a previously published randomized controlled trial. While the original study evaluated treatment effectiveness, the present analysis focused on the responsiveness of outcome measures. No overlapping analyses are reported.

## 3. Results

### 3.1. Participant Characteristics

A total of 77 participants were included in this secondary analysis, with 38 in the active group and 39 in the placebo group.

**Figure 1:**
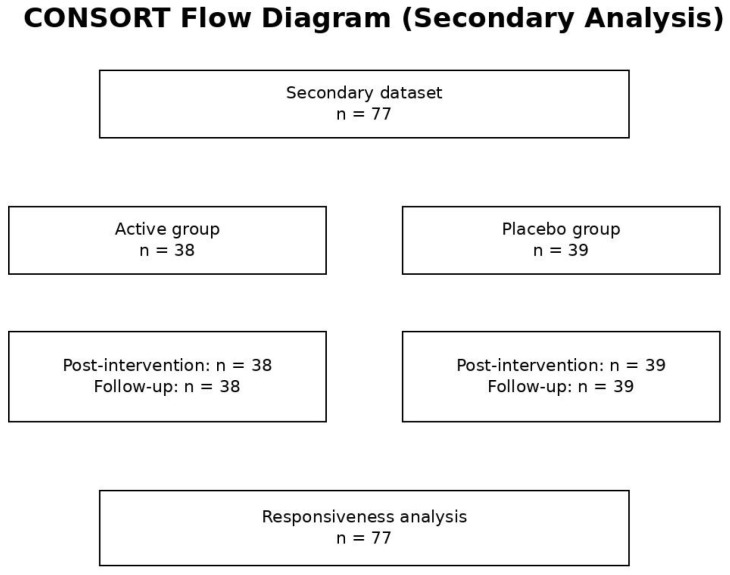
Flow diagram of participant inclusion, allocation, follow-up, and analysis for the secondary dataset according to CONSORT recommendations.

The sample consisted predominantly of female participants, with similar baseline characteristics between groups. Participant flow is presented in Figure 1, and baseline characteristics are shown in Table 1.

### 3.2. Changes over Time

All outcome measures improved at post-intervention (T1). At follow-up (T2), some measures maintained improvements, whereas others showed values closer to baseline.

**Figure 2:**
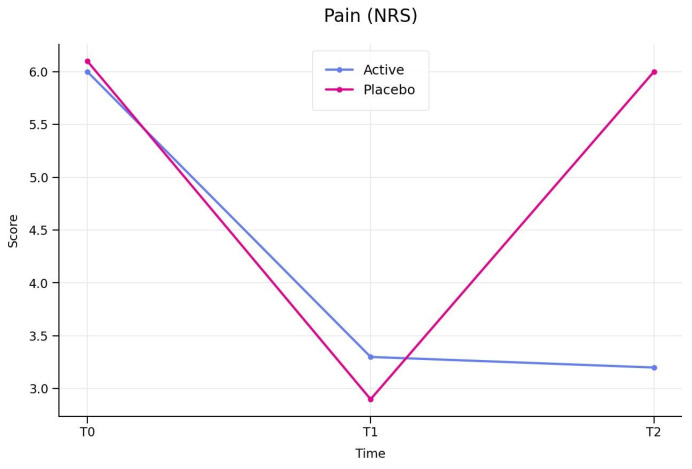
Changes in pain intensity (Numeric Rating Scale) across time (T0, T1, T2) for the active and placebo groups. Values are presented as mean ± standard deviation.

**Figure 3:**
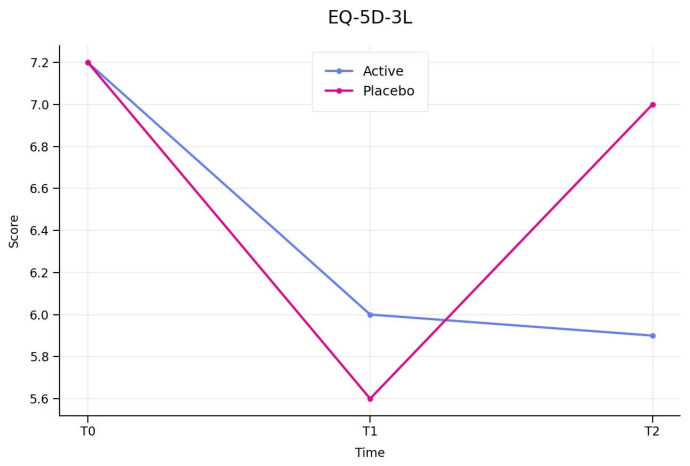
Changes in health-related quality of life (EQ-5D-3L) across time (T0, T1, T2) for the active and placebo groups. Values are presented as mean ± standard deviation.

**Figure 4:**
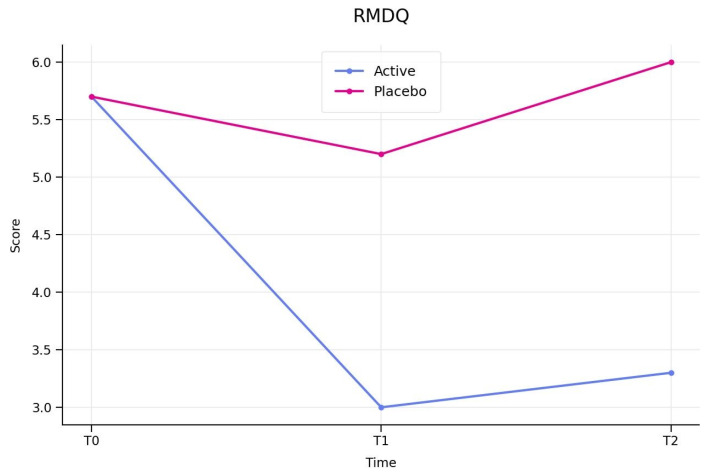
Changes in functional disability (RMDQ) across time (T0, T1, T2) for the active and placebo groups. Values are presented as mean ± standard deviation.

**Figure 5:**
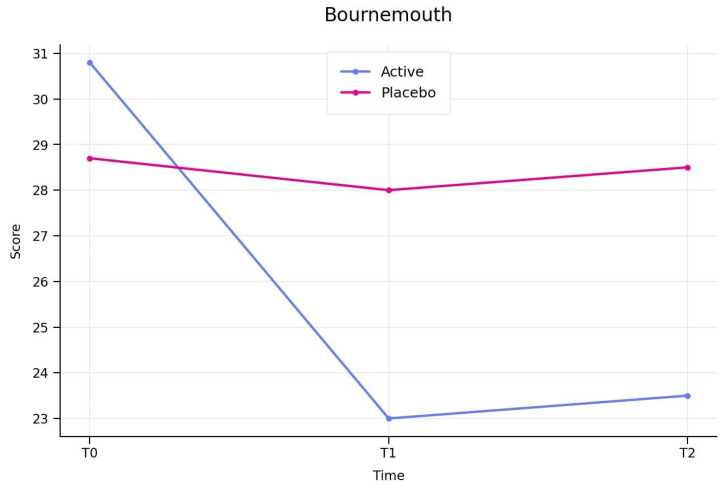
Changes in biopsychosocial impact (Bournemouth Questionnaire) across time (T0, T1, T2) for the active and placebo groups. Values are presented as mean ± standard deviation. Both groups showed changes after the intervention, with greater improvements observed in the active group. The placebo group showed minimal changes and values closer to baseline at follow-up.

**Figure 6:**
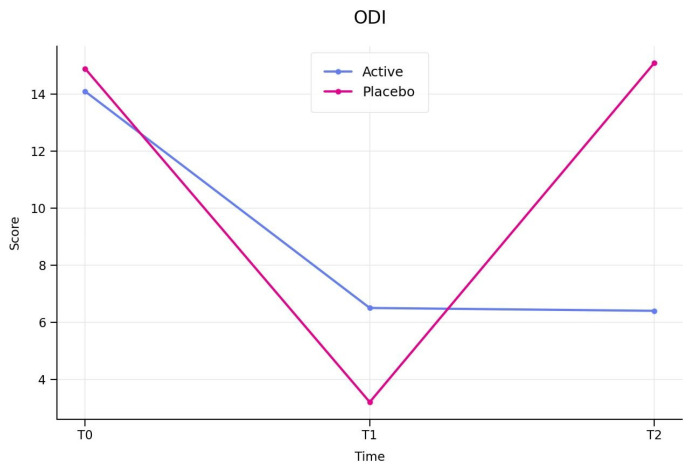
Changes in functional disability (ODI) across time (T0, T1, T2) for the active and placebo groups. Values are presented as mean ± standard deviation.

Descriptive results across time points are presented in Table 2, and temporal trajectories are illustrated in Figure 2, Figure 3, Figure 4, Figure 5 and Figure 6.

### 3.3. Internal Responsiveness

All outcome measures demonstrated the ability to detect change over time.

The largest effect sizes at post-intervention (T1) were observed for pain intensity (NRS: SMD = 1.64, 95% CI: 1.28–2.01) and quality of life (EQ-5D-3L: SMD = 1.08, 95% CI: 0.74–1.41).

Moderate to small effect sizes were observed for functional and biopsychosocial measures (RMDQ: SMD = 0.42; ODI: SMD = 0.16; Bournemouth Questionnaire: SMD = 0.29).

At follow-up (T2), all instruments showed reduced effect sizes compared to post-intervention. Detailed internal responsiveness results are presented in Table 2 and Table 3.

### 3.4. External Responsiveness

External responsiveness analysis demonstrated limited discriminative ability across all instruments.

None of the evaluated outcome measures reached the threshold of acceptable discrimination (AUC ≥ 0.70). The Bournemouth Questionnaire showed the highest AUC values (≈0.63 at T1 and ≈0.62 at T2), followed by NRS and RMDQ, while ODI and EQ-5D-3L presented lower values.

These findings indicate that, although the instruments detected change at the group level, their ability to discriminate clinically meaningful improvement at the individual level was limited. Detailed external responsiveness results are presented in Table 2 and Table 3.

### 3.5. Outcome-Specific Results

All outcome measures showed improvement at post-intervention, with partial maintenance of effects at follow-up.

Pain intensity and quality of life showed greater stability over time, while functional and biopsychosocial measures demonstrated greater variability.

**Figure 7:**
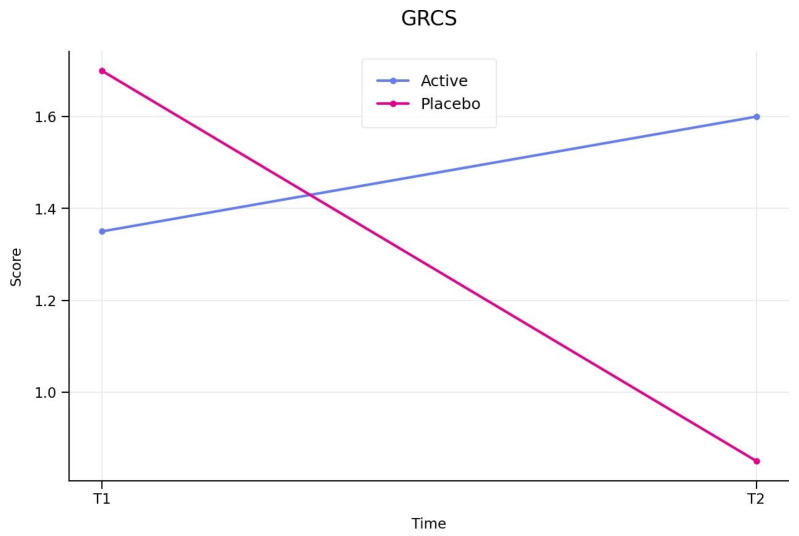
Changes in Global Rating of Change Scale scores across time (T1, T2) for the active and placebo groups. Values are presented as mean ± standard deviation.

Temporal changes for each instrument are illustrated in Figure 2, Figure 3, Figure 4, Figure 5, Figure 6 and Figure 7.

### 3.6. Summary of Findings

The findings of this study indicate that the evaluated instruments demonstrated internal responsiveness ranging from small to large, with the NRS and EQ-5D-3L showing the largest effect sizes. However, external responsiveness was limited across all measures, as none reached the recommended discriminative thresholds.

Among the evaluated instruments, the Bournemouth Questionnaire demonstrated the highest discriminative performance, although still below acceptable levels. Additionally, improvements observed at post-intervention were reduced at follow-up.

## 4. Discussion

This study evaluated the internal and external responsiveness of commonly used outcome measures in individuals with chronic non-specific low back pain using data derived from a randomized controlled trial. Responsiveness is a fundamental measurement property that reflects the ability of an instrument to detect change over time and is considered a key component of longitudinal validity [15].

The main finding of this study was the dissociation between internal and external responsiveness. Pain intensity (NRS) and health-related quality of life (EQ-5D-3L) demonstrated large effect sizes immediately after the intervention, indicating high sensitivity for detecting change at the group level. However, despite these substantial changes, all instruments showed limited ability to discriminate clinically meaningful improvement at the individual level, with area under the curve (AUC) values below the commonly accepted threshold of 0.70.

These findings are consistent with previous studies in chronic low back pain populations, which have reported moderate to large internal responsiveness but limited external responsiveness when anchor-based approaches are applied. This consistency across studies reinforces the challenges associated with identifying clinically meaningful change at the individual level.

The relatively low AUC values observed in this study may reflect limitations of the outcome measures, constraints related to the external anchor, and the inherent heterogeneity of chronic low back pain populations. Additionally, factors such as recall bias associated with the GRCS and variability in individual responses to treatment may have contributed to reduced discriminative performance.

This discrepancy highlights the conceptual difference between internal and external responsiveness. Internal responsiveness reflects statistical change over time, whereas external responsiveness evaluates the extent to which these changes correspond to a meaningful clinical criterion [5,15]. In heterogeneous conditions such as chronic low back pain, where variability in treatment response is high, it is possible to detect group-level improvements without accurately classifying individual-level change.

These findings also raise important methodological considerations regarding the use of responsiveness as an indicator of clinical utility. Instruments that demonstrate high internal responsiveness may still be insufficient for clinical decision-making if they fail to accurately classify individual-level change. This limitation is particularly relevant in clinical settings, where treatment decisions rely on patient-specific outcomes rather than group-level averages.

Another possible explanation for the limited external responsiveness observed in this study is the potential mismatch between the constructs measured by the outcome instruments and the external anchor. For example, the EQ-5D-3L captures overall health status rather than condition-specific improvement, which may reduce its alignment with patient-perceived change. Anchor-based approaches, such as the Global Rating of Change Scale (GRCS), are widely used to interpret clinically meaningful change; however, their performance depends on the chosen cut-off points and classification strategies [5].

Among the evaluated instruments, the Bournemouth Questionnaire demonstrated the highest external responsiveness. This finding is consistent with its multidimensional structure, which incorporates pain, disability, emotional status, and coping-related domains, reflecting the biopsychosocial nature of chronic low back pain [10]. Since patient-perceived improvement is influenced by multiple factors beyond pain intensity, multidimensional measures may be more sensitive to clinically relevant changes. Nevertheless, even the Bournemouth Questionnaire did not reach acceptable levels of discrimination, suggesting that no single instrument is sufficient for individual-level classification.

A reduction in responsiveness was observed over time, with lower effect sizes at follow-up compared to post-intervention. This pattern is consistent with the fluctuating course of chronic low back pain and reinforces the importance of longitudinal assessment when evaluating measurement properties [5].

It is important to emphasize that this study represents a secondary analysis focused on measurement responsiveness rather than treatment effectiveness. Therefore, the results should not be interpreted as evidence of intervention superiority, but rather as an evaluation of the performance of outcome measures in detecting clinical change.

From a clinical perspective, these findings indicate that commonly used instruments are appropriate for detecting change at the group level but have limited utility for identifying clinically meaningful improvement at the individual level. This supports current recommendations that multiple outcome domains including pain, functional disability, and biopsychosocial factors should be assessed to improve interpretation of patient progress [7,8].

The strengths of this study include the use of data derived from a randomized controlled trial, the inclusion of multiple widely used instruments, and the combined evaluation of internal and external responsiveness. However, some limitations should be considered. The relatively short follow-up period may have limited the assessment of long-term responsiveness, and the use of a single anchor (GRCS) may have reduced the sensitivity of external responsiveness analyses.

Future studies should investigate alternative anchor thresholds, the use of multiple external criteria, and longer follow-up periods. Additionally, predictive models may help identify patient characteristics associated with responsiveness, contributing to more individualized assessment strategies.

In summary, the evaluated instruments demonstrated internal responsiveness ranging from small to large, but limited external responsiveness in individuals with chronic non-specific low back pain. These findings highlight the importance of combining outcome measures and using anchor-based approaches to improve the interpretation of clinical change.

## 5. Conclusions

The outcome measures evaluated in this study demonstrated internal responsiveness ranging from small to large, particularly for pain intensity and health-related quality of life, indicating their ability to detect changes over time at the group level. However, this sensitivity was not accompanied by adequate external responsiveness, as all instruments showed limited accuracy in discriminating clinically meaningful improvement at the individual level.

Although the Bournemouth Questionnaire demonstrated the highest discriminative performance among the instruments, its accuracy remained below acceptable levels. These findings suggest that no single outcome measure is sufficient for identifying clinically meaningful change in individuals with chronic non-specific low back pain.

The combined use of multidimensional outcome measures addressing pain, functional disability, and biopsychosocial factors is recommended to improve the interpretation of clinical change and support decision-making in both research and clinical practice.

## Figures and Tables

**Table 1:** Baseline characteristics of the study sample and classification according to responsiveness based on the Global Rating of Change Scale (GRCS).

Characteristics	Total Sample (*n* = 77)	Improved (T1) (*n* = 27)	Not Improved (T1) (*n* = 50)	Improved (T2) (*n* = 35)	Not Improved (T2) (*n* = 42)
Sex (female, %)	68.8%	66.7%	70.0%	74.3%	64.3%
Age (years)	41.37 ± 16.61	37.93 ± 16.00	44.18 ± 16.56	39.77 ± 17.10	—
BMI (kg/m^2^)	25.93 ± 3.72	25.26 ± 3.16	26.30 ± 3.97	26.31 ± 4.61	—

For external responsiveness analysis, participants were classified according to the Global Rating of Change Scale (GRCS). At post-intervention (T1), 27 participants were classified as improved (≥+2) and 50 as not improved (<+2). At follow-up (T2), 35 participants were classified as improved and 42 as not improved.

**Table 2:** Internal and external responsiveness of outcome measures (T0–T1) (*n* = 77).

Outcome	Mean Change ± SD	Effect Size (SMD, 95% CI)	AUC (95% CI)
Bournemouth	−4.53 ± 11.27	0.29 (−0.03 to 0.61)	0.63 (0.50 to 0.76)
ODI	−9.83 ± 10.91	0.16 (−0.15 to 0.48)	0.46 (0.32 to 0.60)
RMDQ	−1.64 ± 2.50	0.42 (0.10 to 0.74)	0.54 (0.41 to 0.68)
EQ-5D-3L	−1.40 ± 1.71	1.08 (0.74 to 1.41)	0.50 (0.36 to 0.64)
NRS	−2.88 ± 2.15	1.64 (1.28 to 2.01)	0.55 (0.41 to 0.70)

Note: SMD = standardized mean difference; AUC = area under the curve; ODI = Oswestry Disability Index; RMDQ = Roland–Morris Disability Questionnaire; EQ-5D-3L = EuroQol 5-Dimension 3-Level; NRS = Numeric Rating Scale. Effect size interpretation: small (0.2), moderate (0.5), large (0.8). AUC interpretation: <0.70 (low), 0.70–0.80 (acceptable), 0.80–0.90 (excellent), >0.90 (outstanding).

**Table 3:** Internal and external responsiveness of outcome measures at follow-up (T0–T2) (*n* = 77).

Outcome	Mean Change ± SD	Effect Size (SMD, 95% CI)	AUC (95% CI)
Bournemouth	−3.85 ± 11.26	0.24 (−0.07 to 0.56)	0.62 (0.49 to 0.75)
ODI	−3.71 ± 7.29	0.29 (−0.03 to 0.60)	0.54 (0.40 to 0.66)
RMDQ	−1.11 ± 2.78	0.27 (0.04 to 0.59)	0.45 (0.31 to 0.58)
EQ-5D-3L	−0.77 ± 1.52	0.45 (0.13 to 0.77)	0.40 (0.27 to 0.52)
NRS	−1.43 ± 2.02	0.68 (0.35 to 1.00)	0.48 (0.35 to 0.60)

Note: SMD = standardized mean difference; AUC = area under the curve; ODI = Oswestry Disability Index; RMDQ = Roland–Morris Disability Questionnaire; EQ-5D-3L = EuroQol 5-Dimension 3-Level; NRS = Numeric Rating Scale. Effect size interpretation: small (0.2), moderate (0.5), large (0.8). AUC interpretation: <0.70 (low), 0.70–0.80 (acceptable), 0.80–0.90 (excellent), >0.90 (outstanding).

## Data Availability

The datasets generated and/or analyzed during the current study are available from the corresponding author on reasonable request.

## Abbreviations

The following abbreviations are used in this manuscript:

CNLBP	Chronic Non-Specific Low Back Pain
OMT	Osteopathic Manipulative Treatment
tDCS	Transcranial Direct Current Stimulation
NRS	Numeric Rating Scal
RMDQ	Roland–Morris Disability Questionnaire
ODI	Oswestry Disability Index
EQ-5D-3L	EuroQol 5-Dimension 3-Level
GRCS	Global Rating of Change Scale
SMD	Standardized Mean Difference
SRM	Standardized Response Mean
AUC	Area Under the Curve
ROC	Receiver Operating Characteristic
